# Improved maximal strength is not associated with improvements in sprint time or jump height in high-level female football players: a clusterrendomized controlled trial

**DOI:** 10.1186/s13102-019-0133-9

**Published:** 2019-09-17

**Authors:** Sigurd Pedersen, Kim Arne Heitmann, Edvard H. Sagelv, Dag Johansen, Svein Arne Pettersen

**Affiliations:** 1 School of Sports Sciences, Faculty of Health Sciences, UiT The Arctic University of Norway, Tromsø, Norway; 2 Department of Computer Sciences, Faculty of Natural Sciences and Technology, UiT The Arctic University of Norway, Tromsø, Norway

**Keywords:** Soccer, Sprint, Counter movement jump, 1RM, Squats

## Abstract

**Background:**

Maximal strength increments are reported to result in improvements in sprint speed and jump height in elite male football players. Although similar effects are expected in females, this is yet to be elucidated. The aim of this study was to examine the effect of maximal strength training on sprint speed and jump height in high-level female football players.

**Methods:**

Two female football teams were team-cluster-randomized to a training group (TG) performing maximal strength training (MST) twice a week for 5 weeks, or control group (CG) doing their regular pre-season preparations. The MST consisted of 3–4 sets of 4–6 repetitions at ≥85% of 1 repetitions maximum (1RM) in a squat exercise. Sprint speed and jump height were assessed in 5-, 10- and 15 m sprints and a counter-movement jump (CMJ) test, respectively. Nineteen participants in TG (18.3 ± 2.7 years) and 14 in CG (18.3 ± 2.4 years) completed pre- and posttests and were carried forward for final analyses.

**Results:**

There was no improvement in neither of the sprint times (*p* > 0.36), nor jump height (*p* = 0.87). The players increased their 1RM in squats (main of effect of time: *p* < 0.00, _p_η^2^ = 0.704), and an interaction effect of time x group was observed (p < 0.00, _p_η^2^ = 0.516) where the TG increased their 1RM more than the CT (between subjects effects: *p* < 0.001, _p_η^2^ = 0.965).

**Conclusions:**

MST improved maximal strength in female football players to a large extent; however, the improvement in maximal strength did not result in any transference to sprint speed or jump height.

**Trial registration:**

This study was registered at clinicaltrials.gov PRS (Protocol registration and results System) with the code NCT04048928, 07.08.2019, retrospectively registered.

## Background

The intermittent nature of football demands complex physiological taxations [1]. Sprint performance seems to be an important factor, which discriminates between competitive level of players where elite female football players sprint faster compared with lower level players [2, 3]. Over the course of a football match, elite female football players sprint (≥25.1 km·h^− 1^) ~ 200 m, distributed in ~ 30 bouts, of which 95% are sprints under 10 m [4], and interestingly, the speed of the sprints has increased for female football players over the last two decades [5], emphasizing the growing importance of sprints in female football.

Approaches for improvements in sprint are many, including sprint training, explosive movements and strength training (ST) [6–8]. Traditionally, ST regimes for developing speed and explosiveness have mainly consisted of repetitions of high velocities and low loads [9]. However, training regimes consisting of training with high loads and low velocity repetitions, usually between 3 and 5 sets of 4–6 repetitions ≥85% one repetition maximum (1RM) has emerged as a supplement and/or replacement to the low-load high velocity training [10]. The high load low velocity strength training, usually expressed as maximal strength training (MST), is effective for improving maximal strength [11], and may also result in improvements in muscle power and rate of force development in male football players [12]. The rationale behind this training modality for improvements in explosive actions builds on the significant relationship between 1RM and movement velocity, sprint performance and jump height [13, 14]. In contrast to the principle of training specificity, training of maximal strength in a nonspecific movement tempo combined with the specific movement itself, is more effective than just training the fast movement alone [15]. The effect from MST on power actions could be explained by an improved neural drive to the muscles involved [16, 17] due to the training being performed with maximal intended velocity combined with a load approaching the upper limits of motor unit recruitment [18].

Moreover, the goal of ST is often increased muscle mass [19], however, when comparing conventional hypertrophy training (60–70% of 1RM, 8–12 repetitions) with MST, MST is superior concerning gains in both 1RM and rate of force development [11, 20]. Additionally, an increased muscle mass may be detrimental for sports performance involving endurance, such as football, due to the increased body mass. Thus, improving strength with minimal hypertrophy should be favourable, as this will lead to an increased relative strength. According to Newton’s second law of motion, an increased relative strength should improve jump height and sprint speed. Minimal hypertrophy in relation to maximal strength gains is best achieved by ST with high loads and low volume [11, 20–22].

In male football players, studies have reported a favorable effect on both 1RM, sprint and jumping performance following MST [20, 23]. Although females and males possess diverse levels of anabolic hormones, they do in general respond similarly after training interventions in most strength outcomes [24]. However, there are reports of a larger relative increase in females compared to males when the same ST is applied [25]. Further, on the muscle fiber level, heavy resistance training is reported to induce hypertrophy for type IIX fiber cross-sectional area in young males only, when compared with young females [26], indicating potential gender differences in response to ST. The effect on strength, sprint and jump height performance following MST in female football players is still to be elucidated. Thus, the aim of this study was to examine if improvement in maximal strength is associated with improvements in sprint and jump height performance following MST.

## Methods

In this cluster-randomized controlled trial, two football teams (playing at level 2 and 3 in Norway) was invited to participate. The study were conducted during the last part of the pre-season preparations, ending 1 week before first seasonal competition. The training group (TG) performed MST training carried out as free-barbell squats twice a week over 5 weeks in addition to the planned pre-season training, while the control group (CG) was instructed to perform their originally planned pre-season training.

### Subjects

The total sample comprised 46 players aged 15–26 years, where two separate football teams were cluster-randomized to either TG or CG (Table 1). The two teams played at level two and three in Norway, where level two is a national league and level three a regional league in Northern Norway. Inclusion criteria was that the players perceived themselves as injury free and able to complete the strength training. Randomization was carried out using the online tool http://www.randomlist.com/team-generator by the first author. Players were only excluded if having injuries that made strength training, running and jumping unachievable. The players carried out ~ 6.5 h training per week with their team (Table 2). Four players were injured, two did not complete the required amount of training, one withdrew due to time limitations and five withdrew without providing any reason resulting in 19 participants in TG and 15 in CG that completed both pre- and posttests, and were included in the analyses for training effect (Table 3).

**Table 1:** Characteristics of participants

	TG (*n* = 24)	CG (*n* = 22)
Age (years)	18 ± 3	19 ± 2
Body mass (kg)	62 ± 6	63 ± 10
Height (cm)	167 ± 6	168 ± 5
BMI (Kg/m^2^)	22.1 ± 2	22.3 ± 3
Experience with the squat exercise		
None	3	8
Some (< 1 year)	14	9
Much (> 1 year)	7	5

Data are mean ± standard deviation. *TG* Training group, *CG* Control group, *BMI* Body mass index

**Table 2:** Weekly team training for CG and TG prior to inclusion

	CG	TG
Sessions (n)	4–5	4–5
Passing, technique, finishing, possession (min^−1^)	60	270
High intensity small sided games (min^−1^)	90	90
Running and conditioning (min^−1^)	90	45
Strength, balance and injury prevention (min^−1^)	90	0
Stretching (min^−1^)	60	0
Total training time (hours^−1^: min^− 1^)	6:30	6:45

According to the declaration of Helsinki, all participants were fully informed of the potential benefits and risks of the study, both orally and written, before signing an informed consent. For participants under 16 years, both the players and their parents gave their written informed consent. The participants were fully informed of their rights to withdraw from the study at any time without providing any reason. This study was approved by the Norwegian Centre for Research Data for the storage of personal data (Approval reference number: 59063 / 3).

### Procedures

All testing and training sessions were conducted in an exercise training laboratory at Alfheim Stadium, Tromsø. Prior to the intervention, the players underwent baseline tests over two test days, with a 72 h washout period to avoid any detrimental effects from the preceding test day: day 1) measurement of body mass and body height, 5-, 10- and 15 m sprint time and a counter-movement jump (CMJ), day 2) 1RM in a free-barbell squat exercise with partial 90° knee angle range of motion (ROM).

Prior to the tests, the participants were asked to refrain from heavy training the preceding day, and to arrive in the laboratory well-hydrated. All tests and training sessions started in the afternoon, with the same general warm-up routine: 7 min of self-selected low intensity cycling on an ergometer bike (Pro/Trainer, Wattbike Ltd., Nottingham, UK) followed by 7 min low intensity running of self-selected speed on artificial grass.

On day 1, following the general warm-up and three 15 m strides on a sprinting field, a 15-m sprint test was carried out. Data were assessed in 5 m splits by photocells mounted to the floor and walls (ATU-X, IC control AB, Stockholm, Sweden) using single-beam electronic barriers. The within-subject coefficient of variation is 2% for this measurement [27]. The surface consisted of artificial grass, and the players wore their own running shoes. The sprints started with the players in a static position placing their front foot 30 cm behind the starting line. A timer was triggered by the participant breaking the initial sensor. The rest interval between the single sprint trials was 180 s [10]. The fastest sprint time of three trials was carried forward for further analyses.

Thereafter, the players rested for 5 min prior to performing the CMJ test [23]. CMJ was assessed by a portable force platform (Hur-Labs, ALU4, Finland), with a validity within 1 cm (2%) when compared with the gold standard mounted floor force platform [28], and a within-subject coefficient of variation of 2.8% [29]. Force data were recorded by a software (Force platform software suite, HURlabs oy, Kokkola, Finland). This device records only the vertical ground reaction force at a sampling frequency of 1200 Hz and jump height is automatically calculated by software applying double integration of the force signal through Simpson’s rule of integration. The players were instructed to keep their hands placed on the hips and the feet shoulder-width apart. Each player performed two trials with a ≥ 180 s rest between sets. The highest jump was carried forward for further analysis. Day 1 was ended with a familiarization trial for the squat exercise with low loads.

On day 2, the players returned to the laboratory for the assessment of maximal strength as 1RM. The session was initiated with the same general warm up routine as mentioned above. An Olympic barbell (T-100G; Eleiko, Halmstad, Sweden) and a suitable rack was applied for testing of 1RM. The ~ 90° knee angle of each participant was measured during every repetition using a goniometer, and the players were given an orally “go” when being allowed to start the concentric phase of the lift. Prior to starting their 1RM attempts, the participants warmed up with 10 repetitions with a low load of ⁓ 50% 1RM (subjectively assessed by the instructor). The starting 1RM attempt was an initial acceptable load decided by the instructor. Each 1RM attempt was carried out by a single repetition, with increasing load of 5–10 kg until they failed to execute the 1RM attempt, which on average was five trials. Each attempt was interspaced by ≥180 s of rest. The within-subject coefficient of variation for squat 1RM is 2.9% [30].

### Training intervention

**Fig. 1:**
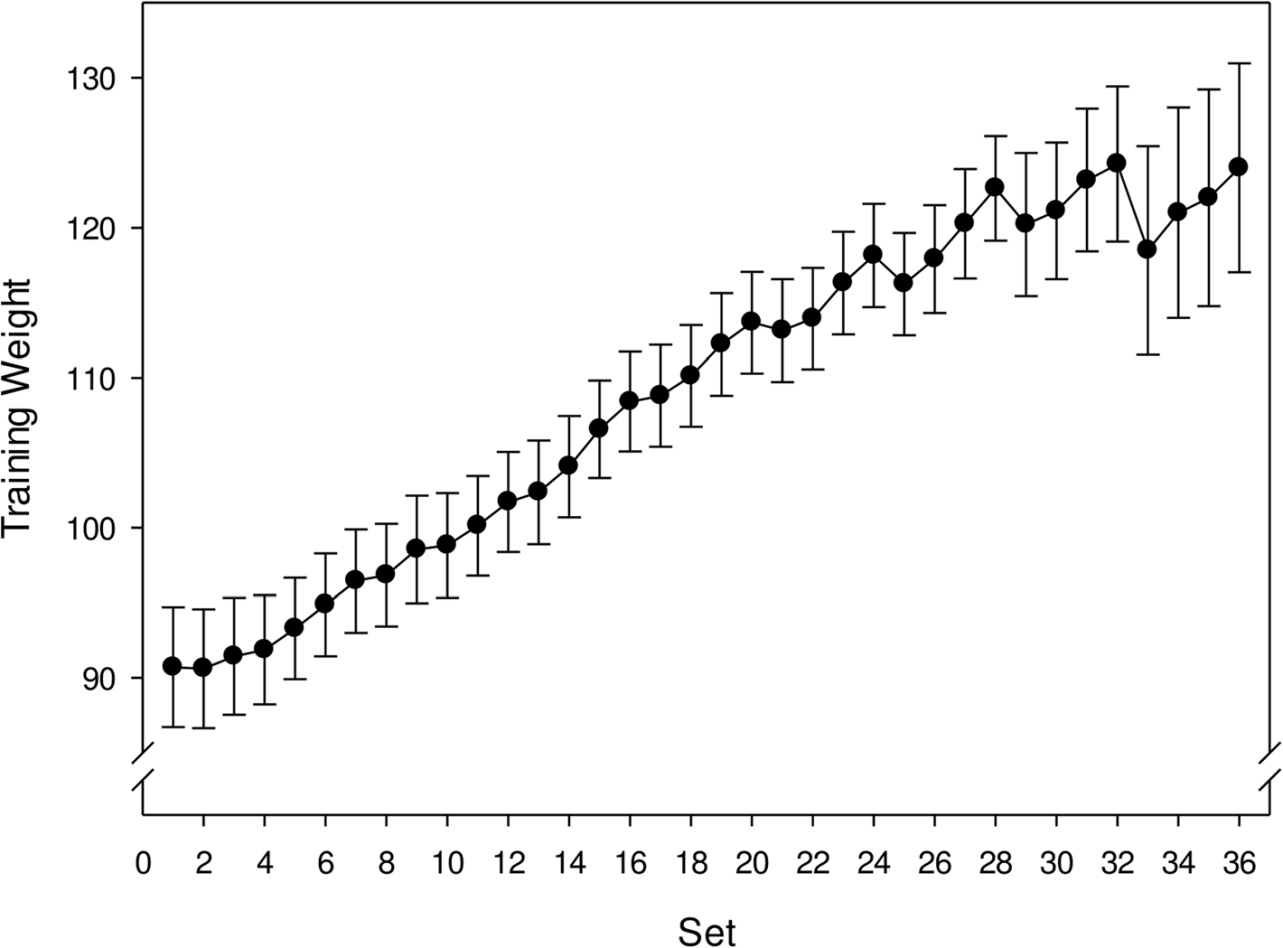
The logged training for the 90° squat exercise performed as maximal strength training (MST) by the training group. The dots represent the average weight lifted ± SE (vertical bars) during each set

The players attended supervised training in the laboratory twice a week for 5 weeks. The training session started with the general warm-up routine described above, before starting the strength training. The program consisted of 90° squats, carried out in the same way as in the 1RM test. The squat training was initiated with three sessions of three sets of six repetitions, followed by seven sessions of four sets of four repetitions. The repetitions were carried out with a slow eccentric movement followed by maximal mobilization in the concentric phase. One hundred eighty second of recovery was given between each set [23]. The load was initially set at 85% of pre-test 1RM, which the participants increased with 2.5–10 kg if they could manage more than six or four repetitions, depending on their scheduled program, resulting in a consistent overload during the whole intervention (Fig. 1). Weight lifted for each repetition was logged continuously during the study. Additionally, for ethical reasons, in order to avoid hamstring strains due to an anticipated large agonist-antagonist strength ratio following the intervention, three sets of six repetitions of the Nordic hamstring exercise were performed after the squat exercise for each session with a ≥ 180 s rest period between sets [31] (Fig. 1).

### Statistical analyses

All statistical analyses were carried out using the Statistical Package for Social Sciences (SPSS, Version 25, IBM, USA). The Shapiro Wilk test confirmed all data, except for body weight in CG (*p* = 0.02) and 5 m sprint in TG (p = 0.02), to not deviate from normal distribution. Data were analysed via a two x two repeated measure analysis of variance (ANOVA). Two levels corresponding to the groups (i.e., TG and CG) are specified as the between-subjects factor. The within-subjects factor (time of test) represents the pre- and post-tests. Effect sizes were calculated as partial eta squared (_p_η^2^) were a small, medium and large effect size was determined as 0.01–0.05, 0.06–0.13 and ≥ 0.14 _p_η^2^ [32]. All values are presented as mean ± standard deviation (SD). Descriptive values for female football players in 90° squat is reported once (112 kg) [33], where SD is not reported. In one similar study in males where mean 1RM squat strength is 116 kg, the reported SD was 20.1 [23] kg. It is previously reported that a 24% increase in 1RM squat strength results in a 2% improved sprint performance in male football players [8]. Thus, to observe a 24% improvement in strength with 80% power and an alpha level of 0.05, 9 participants are required in each group.

## Results

Nineteen participants in the TG and 15 participants in the CG performed all pre and post-tests and ≥ 70% of all training sessions (one subject performed 70%, seven subjects performed 80%, five subjects performed 90% and six subjects performed 100%). The baseline values for the participants included in the intervention analysis were not different between the two groups (Table 3).

**Table 3:** Effect of training on body mass, physical performance measures and strength derivatives (Mean ± SD)

Variables	TG (*n* = 19)		CG (*n* = 15)		*P*-value*
	Pre	Post	Pre	Post	
Body mass (kg)	61.67 ± 5.40	62.18 ± 5.31	62.92 ± 10.48	64.09 ± 10.49	0.13
Sprint time (s)					
5 m	1.06 ± 0.05	1.05 ± 0.05	1.06 ± 0.06	1.07 ± 0.06	0.07
10 m	1.89 ± 0.07	1.89 ± 0.08	1.90 ± 0.09	1.90 ± 0.09	0.74
15 m	2.66 ± 0.10	2.64 ± 0.12	2.67 ± 0.13	2.66 ± 0.13	0.51
CMJ Jump Height (cm)	27.32 ± 4.94	27.19 ± 5.93	25.82 ± 5.45	26.12 ± 4.83	0.65
1RM 90° squat (kg)	106 ± 21	137 ± 16	118 ± 28	124 ± 31	0.00
1RM 90° squat (kg/ m_b_ kg^− 1^)	1.73 ± 0.33	2.21 ± 0.32	1.88 ± 0.34	1.94 ± 0.37	0.00
1RM 90° squat (kg/m_b_^-0.67^)	6.81 ± 1.29	8.73 ± 1.14	7.45 ± 1.39	7.73 ± 1.52	0.00

*TG* Training group, *CG* Control group, *CMJ* Counter movement jump, *1RM* 1 repetition maximum. *P-value represents between subjects effect

There was no main effect of time for 5 m (*p* = 0.77, _p_η^2^ = 0.003), 10 m (*p* = 0.82, _p_η^2^ = 0.002) or 15 m (*p* = 0.36, _p_η^2^ = 0.026) sprint time, and consequently no interaction effects of time x group was observed (5 m: *p* = 0.72, _p_η^2^ = 0.097, 10 m: *p* = 0.74, _p_η^2^ = 0.003, 15: *p* = 0.51, _p_η^2^ = 0.014) (Table 3).

Similarly, no main effect of time for CMJ was observed (*p* = 0.87, _p_η^2^ = 0.001), and consequently no interaction effect of time x group was observed (*p* = 0.65, _p_η^2^ = 0.006).

The players increased their 1RM in squats (main of effect of time: *p* < 0.00, _p_η^2^ = 0.704), and an interaction effect of time x group was observed (*p* < 0.00, _p_η^2^ = 0.516) where the TG increased their 1RM significantly more than the CT (between subjects effects: *p* < 0.001, _p_η^2^ = 0.965).

**Fig. 2:**
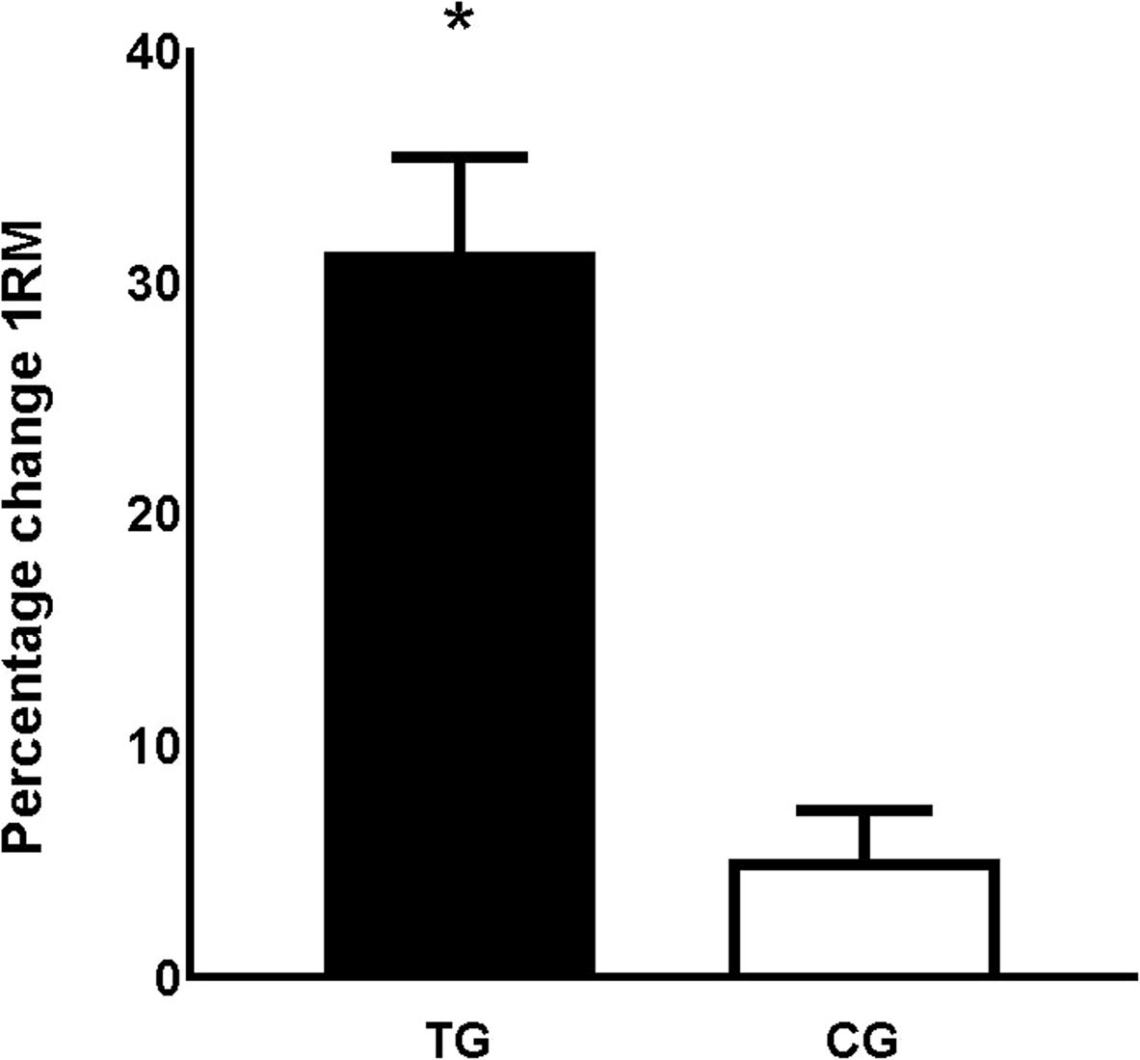
The mean percentage change from pre- to posttest for 1RM ± SE in TG and CG. TG = training group; CG = control group; 1RM = 1 repetition maximum. * = Between group difference *p* < 0.01

The players increased their body mass (*p* < 0.001, _p_η^2^ = 0.332), however, no interaction effect between groups was observed (*p* = 0.13, _p_η^2^ = 0.070) (Fig. 2).

## Discussion

In this cluster-randomized controlled trial, 5 weeks of MST improved 1RM, but this maximal strength improvement did not result in any improvements in sprint time or CMJ performance in female football players.

We observed a large increase in absolute 1RM strength of 31 kg (31%) for the TG, being highly superior to the 5% increase in 1RM for the CG. This is a more pronounced increase in strength than observed on average for highly trained male football players [8]. On the one hand, as untrained individuals seem to have greater improvements in strength following ST compared with trained individuals [34], the large improvements in this study may be due to the low experience of ST in the participants of the present study. On the other hand, the baseline values in this study was similar to previously reported values for elite female football players [33], where the present study’s participants ended up being considerably stronger than their elite peers (the present study’s participants: 137 kg, previously reported values for elite peers: 113 kg). Moreover, following 5 weeks of MST, the participants in our study displayed similar absolute 1RM 90° squat strength (present study: ~ 136 kg) as two previously reported elite male football teams (Male players: ~ 116/ 135 kg,) [15, 23].

Previous studies applying ST in female football players have assessed strength outcomes in isometric [35] or isokinetic exercises [36, 37], making comparisons with the present study unattainable as we measured dynamic squat strength [38]. Elite male football players experienced a 52% increase in absolute 1RM following 16 sessions of MST over 8 weeks [23]. Considering the similar relative increase per training session in the present study (~ 3.6% increase per session) compared with the study by Helgerud et al. [23] (~ 3.2% per session), one may speculate that there are small sex differences in strength improvements following MST in football players. As a linear increase in strength gain has been observed from onset of ST with up to 8 weeks before sign of plateau is observed [39] and duration of our study was 5 weeks, one may speculate whether the players in our study did not reach their expected plateau for strength improvements.

The present study’s participants displayed an increase in body mass, which is consistent with earlier findings in men [23]. Considering that both the TG and the CG in the present study performed pre-season training, one explanation for the increased body mass may be an increased water uptake in muscles due to the improved glycogen uptake in muscles [40], which is observed following training initiation [41]. Nevertheless, it is unlikely that the increased body mass solely explains the large increase in 1RM strength in the present study, suggesting a large improvement in neural drive and/or improved motor unit recruitment following the MST intervention [39].

There were no improvements in sprint following MST in this study, which is in contrast to previous findings in male football players [20, 23]. In fact, an average increase of 23.5% increase in 1RM is required for a 2% improvement in 10 m sprint in the males [8]. The previous studies conducted in males employed longer intervention duration compared with this present study [10, 20, 23]. Thus, as there may be a dose-response relationship between improvements in explosive actions and ST training duration [42], the intervention duration may have been too short in the present study [43]. However, there are other possible explanations for the lack of improvement in sprint performance. One may be that the players performed insufficiently amounts of specific sprint training in the pre-season cycle. It is previously shown that in order to improve sports-related high-velocity movements, these movements must be performed in everyday training [44]. Moreover, as football is concurrent sport with need for both endurance and strength, an interference effect could be present for the adaptation to training [45]. Interestingly, the interference effect from concurrent training is shown to be more pronounced for adaptations to power actions, compared with adaptations to strength, meaning that force at high velocities is affected to a larger extent than force at low velocities [46], which could explain the lack of improvement in sprint and jump abilities, although strength was increased.

There were no jump height improvements in the present study, which is consistent with a previous study in females [47], but in contrast to a study conducted with males [8]. Previous studies who observed an increased CMJ jump height in female football players included plyometric training [42, 48–50], thus, as for sprint adaptations, the specificity of training may explain the unimproved jump performance as well [44].

A stronger muscle will tolerate a higher force, making it more resistant to injury. Although not measured in the current study, ST reduces injury rate, and shortens rehabilitation time [51]. Moreover, the strength of connective tissues and joint stability is also improved after ST [52]. That is of particular importance in the female football player population as they have a two to six times greater prevalence of anterior cruciate ligament injuries compared to their male equivalents [53]. The players in the present study showed similar body mass values as their national level peers [53], which is suggested as optimal for physical performance for female football players [54]. Moreover, as age, anthropometry and physical variables did not differ between the two teams in the present study, comparisons were regarded as appropriate. Finally, although performance by means of sprint speed or jump height was unchanged in the current study, an increased strength could potentially improve performance through pathways not assessed in this paper. For example, this could lead way for future studies with higher ecological value, where the assessment of fatigue delay, technical actions and number of interceptions during match play could be studied following such a large increase in strength. MST has mainly been evaluated in male athletes, and this study is to our knowledge the first to investigate the effect of MST for dynamic strength 1RM, sprint speed and jump height in female football players.

There are several limitations of this study. First only six of the participants accomplished 100% of the training, which means that the other participants had some weeks when they only performed one ST session. Moreover, the CG was instructed to continue their habitual training, without any substitute for the added training time seen in the TG during the intervention. Thus, we are not able to distinguish between the effect of MST and the effect of an added training volume per se.

Although the participants in this study played at senior level football, their mean age was ~ 18 years, which can be considered junior level age. In contrast, those who are competing at the national level and in the highest level domestic leagues in other countries, are typically between 20 and 27 years [53]. Thus, although the participants in the present study aim at competing at the highest level possible, they may not be at the peak of their performance level at the present age. Secondly, we propose improved neural adaptations as the main mechanism driving the observed increase in 1RM. However, more sophisticated measurement methods are required to assess which type of neural adaptations that are responsible for this increase.

## Conclusions

MST improves maximal strength in female football players; however, the improvement in maximal strength did not result in any transference to improvements in sprint speed or jump height in this study. This indicates that female football players may need to incorporate specific sprint and jump training into their weekly training routines in order experience improvements in sprint and jump performance. However, our intervention was only 5 weeks, hence, it is unknown whether a longer intervention period would allowed these players to improve their sprint and jump performance, as it is observed previously for male players.

## Data Availability

Trial protocol and the datasets used in in the current study are available from the corresponding author on reasonable request.

## Abbreviations

1RM: 1 Repetition maximum
CG: Control group
CMJ: Counter movement jump
MST: Maximal strength training
TG: Training group
